# Design and User Experience of VirNE Application: Deep Breathing Exercise in a Virtual Natural Environment to Reduce Treatment Anxiety in Pediatrics

**DOI:** 10.3390/healthcare11243129

**Published:** 2023-12-09

**Authors:** Ilmari Jyskä, Markku Turunen, Arash Chaychi Maleki, Elina Karppa, Sauli Palmu, John Mäkelä, Kaija Puura

**Affiliations:** 1 TAUCHI Research Center, Faculty of Information Technology and Communication Sciences, Tampere University, FI-33014 Tampere, Finland; markku.turunen@tuni.fi (M.T.); arash.chaychi@gmail.com (A.C.M.); john.makela@tuni.fi (J.M.); 2 TamCAM Research Center, Faculty of Medicine and Health Technology, Tampere University, FI-33014 Tampere, Finland; elina.karppa@gmail.com (E.K.); sauli.palmu@tuni.fi (S.P.); kaija.puura@tuni.fi (K.P.); 3 Department of Pediatrics, Tampere University Hospital, Central Hospital, P.O. Box 2000, FI-33521 Tampere, Finland; 4 Department of Child Psychiatry, Tampere University Hospital, Central Hospital, P.O. Box 2000, FI-33521 Tampere, Finland

**Keywords:** virtual reality, child, anxiety, stress, nature, analgesia, virtual natural environments, deep breathing, user experience

## Abstract

Treatment anxiety is a serious problem among child patients. A few studies have addressed this issue with virtual reality solutions, with promising results; however, the applications used have generally been designed for entertainment instead of this purpose. This article studies the potential of using deep breathing exercises in a virtual natural environment to address this issue, with a focus on design approach and user experience. It presents the VirNE—Virtual Natural Environments relaxation application, which is based on known stress-reduction methods, and a feasibility study conducted with it in a local hospital. The study had a within-subjects design, and it included 21 eight to twelve-year-old child patients, who used the application during an intravenous cannulation procedure related to their treatment. The study found good user acceptance and user experience both among the child patients and pediatricians, with the perceived usefulness of the method being higher among the patients with increased levels of anxiety or needle phobia. In addition, a clear stress-reducing effect was found. This offers proof-of-concept for the multidisciplinary design approach based on existing scientific knowledge regarding the desired effect for pediatric virtual reality applications for this use context.

## 1. Introduction

Experiences of stress in the form of pain and anxiety during invasive procedures are a significant problem among child patients. As a consequence, possible negative effects include extreme physical reactions during the procedure and major anxiety towards future medical procedures, which in turn can lead to avoidance of treatment in later life [1,2]. Recent studies [3,4,5,6] have shown that modern virtual reality (VR) solutions have good potential to address this issue, but the number of studies with child patients remains limited. While studies generally support the idea of using VR methods in pediatrics for pain and anxiety reduction, there is an ongoing scientific debate regarding what kind of VR content should be used in this use context, with the discussion revolving around the idea of distraction by VR and the question of whether the VR contents should be passive or interactive. This approach can be somewhat misleading, as it over-simplifies the nature of VR content—VR applications can simultaneously have both passive and interactive elements, and when using a head-mounted display (HMD), even the most passive VR content usually involves system output that resembles interactivity in the form of the visual view being controlled by user’s head movements, similar to real life.

This article studies the potential of a novel VR relaxation method that combines both passive and interactive VR elements for reducing the stress and anxiety of eight to twelve-year-old child patients during an intravenous cannulation procedure, which includes a stress-inducing penetration of the skin of the patient with a needle. This VR relaxation method focuses on scientifically proven anxiety reduction methods, namely, deep breathing [7] and experiencing a relaxing natural environment [8], rather than plain distraction by VR or the amount of interaction with the VR content.

A feasibility study was conducted in a local hospital with 21 child patients who received cannulation while using a HMD for experiencing a deep breathing exercise in a virtual natural environment. The study found positive results in terms of anxiety reduction and user experience. This article focuses on the user experience and the design process of a VR application featuring this relaxation method. In other words, detailed findings regarding the effect are not included in this article.

However, another article by the authors [9] focuses on the effective qualities of using deep breathing exercises in a virtual natural environment in a pediatric context. Key findings regarding the effects of this method include strong objective evidence of a clear stress-reducing effect regardless of the level of general anxiety or the level of needle phobia of the patient. This statistically very significant effect was found from heart rate variability (HRV) [10,11] measurements of the study participants by comparing baseline and VR intervention (pre-cannulation) values of all four stress-related HRV variables used in the study. No adverse effects were found. However, the subjective evaluations of the effect collected with VAS-A questionnaires [12,13] had more variance, with the results showing increased stress reduction among patients with higher levels of needle phobia.

## 2. Background

To alleviate the fear of medical procedures or needles, strategies such as distraction or educating the patient about the upcoming situation have been employed [14]. The treatment may include sedative medications, with Midazolam, propofol, or dexmedetomidine being the most common choices. However, it’s important to note that the use of sedatives poses a risk of adverse effects for the patient [15].

The use of VR methods for anxiety reduction in healthcare services offers a non-pharmacological and non-invasive alternative to more traditional methods [16,17,18]. VR methods have also been successfully used to alleviate pain and distress related to the treatment [19,20], and are generally well-received among both child patients [21] and healthcare professionals [22]. Other benefits of VR methods include cost-effectiveness and economy of scale [23,24], repeatability, and a low risk of adverse effects [25]. Challenges include a need for true multidisciplinary collaboration when developing therapeutic VR applications [26], a relatively low level of related technical skills among the medical staff, and the resulting need for training and technical support for the use of VR equipment [22,27]. In addition, the possibility of nausea due to cybersickness [28,29], and the likely gradual decrease in novelty value in repeated use [27] needs to be considered. Furthermore, VR technology can be used to digitally replicate known stress-reduction methods in a treatment scenario where these methods would otherwise be too resource-demanding, impractical, or plain impossible to conduct in pediatric care—for example, the positive effects of being in a pleasant forest environment [30].

Recent studies suggest that the use of virtual natural environments, which refer to digitally replicated natural environments, is likely to cause a positive effect on stress and anxiety reduction, which is similar to the effect of real natural environments [31,32,33,34]. Although the majority of related studies seem to have a focus on virtual forest environments, similar positive effects have been found in studies regarding other virtual environments too—for example, virtual natural environments with water landscapes also seem to be efficient in producing stress relief [35,36]. Furthermore, based on their positive findings regarding virtual desert environments, Yin et al. suggest that past experiences and familiarity play a role in the health benefits of these virtual natural environments [37]. One of the benefits of virtual natural environments is the possibility to use them in combination with other relaxation methods.

Deep breathing is a common, effective, and very cost-efficient method for reducing stress and anxiety levels acutely while supporting emotional self-regulation [7,38], but it is not currently widely used in pediatric care, which tends to resort to medication for similar purposes [39]. This may be partially related to practical and treatment consistency issues, including a lack of validated procedures for deep breathing in pediatrics. Even so, an increasing amount of scientific evidence supports the idea of using deep breathing exercises in virtual natural environments to effectively reduce stress and anxiety [40,41,42], although research with patients in pediatric care remains very limited.

Based on this scientific background, the authors designed a therapeutic VR application based on virtual natural environments and deep breathing guidance. The aim was to study whether this method could offer a new tool for pediatricians to reduce anxiety and fear of treatment in child patients by measuring both the effect [9] and user experience of it in a real treatment scenario.

## 3. Virtual Natural Environments (VirNE) Application

Virtual Natural Environments (VirNE), the VR application used in this research, is a relaxation application that aims to reduce the stress and anxiety levels of the users. The application combines 360-degree videos of natural environments with guided relaxation exercises, and it is used with a Meta Quest 2 HMD (Reality Labs, Redmond, WA, USA) [43], which is remote-controlled by a computer running the software with Oculus Link [44]. This remote-control feature allows the nurse using the application to control the exercise attributes and provides necessary information about the designated timeframe for performing the cannulation procedure while hiding this potentially anxiety-inducing information from the child patient.

The target user group of the application is eight to twelve-year-old children experiencing stress and anxiety. In this study, VirNE was used to deliver the deep breathing exercise in a virtual natural environment with the aim of reducing treatment anxiety in invasive pediatrics.

### 3.1. VirNE Development in Brief

VirNE development was conducted between summer 2021 and spring 2022. It was developed by a multidisciplinary team consisting of child psychiatry, computer science, media, and human–computer interaction experts. The application is based on a previous prototype [31], and the development process was based on recommendations by Simonetti et al. regarding pediatric VR application development [5].

### 3.2. Exercise Design

The deep breathing exercise used in the study is based on dialog guidance recorded in one session with a voice actor. The dialog was directed by a physician and recorded by a media professional. A motherly tone targeted at children was used. The scripts for the exercises were written by the physician, with minor revisions by the rest of the team.

**Figure 1:**
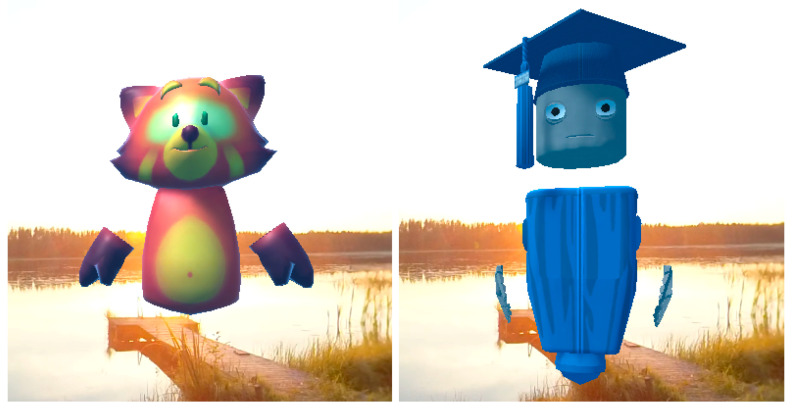
VirNE avatar characters used in the research: The Fox (**left**) and the Bot (**right**). The third option was to use the application without a visual representation of the exercise guidance dialog.

The application includes a visual presentation of the exercise dialogues in the form of an optional avatar character. This avatar character can be chosen from two different options, a Fox character and a Bot character, which are based on free avatar characters from Mozilla hubs [45] and are presented in Figure 1. This avatar character is stationary in the application, hovering slightly above the ground level in the virtual environment, close to the default point of view of the user, and slightly above the vertical location of the HMD. The exercise dialog audio is used to animate the character’s head—more precisely, the nose of the fox and the mouth of the bot move in the rhythm of the dialog. The exercises can also be run without a visual avatar character.

**Figure 2:**
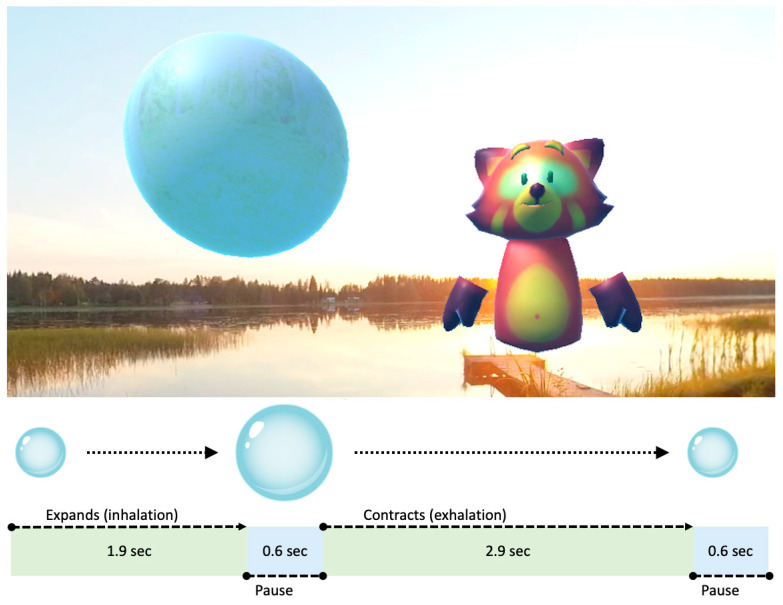
The breathing balloon animation and the Fox character in a VirNE exercise, along with an explanatory breathing animation diagram.

An animated balloon is used to guide the breathing rhythm during the exercise, which expands and contracts in the rhythm of the desired breathing cycle. According to Zaccaro et al. [38], the optimal breathing frequency for adults is six breaths per minute which is about half of the usual breathing frequency. Using this principle, for eight to twelve-year-olds, the optimal breathing frequency was calculated to be 10 breaths per minute. The research version of the application used a six-second breathing cycle with a 1.9 s inhale period and a 2.9 s exhale period, both followed by a 0.6 s pause in between. Gentle inhalation and exhalation sounds are played along with the animation accordingly to support breathing awareness. Figure 2 illustrates this functionality, presenting both the default point of view and the breathing balloon animation cycle.

The balloon initially appears slightly to the left of the default point of view of the user and horizontally slightly above the location of the HMD. After that, it slowly follows the user’s point of view when it is over 90 degrees away from the center of the user’s point of view.

### 3.3. Exercise Structure

The deep breathing exercise aims to reduce stress and anxiety by helping the user to breathe in a slow and deep rhythm. The total length of this exercise program is 6 min, and it is based on dialog guidance presented by an avatar character and supported by a looping breathing balloon animation. The structure of the exercise is presented in Table 1.

### 3.4. 360-Degree Video Material

**Figure 3:**
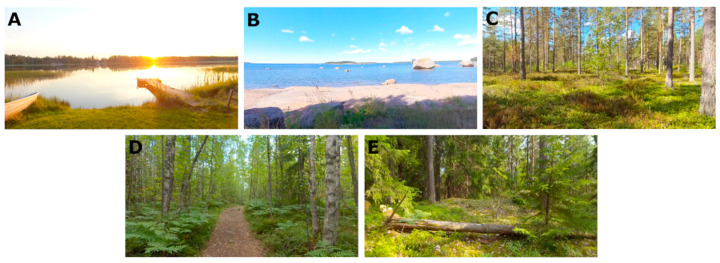
The five VirNE natural environments used in this research: (**A**) Sunset by a lake shore in the Pirkanmaa region; (**B**) Ocean shore in the Kymenlaakso region; (**C**) Dry coniferous forest in the Kymenlaakso region; (**D**) Path in a deciduous forest in the South Karelia region; (**E**) Coniferous forest in the South Karelia region.

For this application, 360-degree video material for the virtual natural environments was recorded with an Insta360 Pro camera (Insta360, Shenzhen, China) in several calm Finnish natural environments, using a camera stand that positioned the camera at a height of 120 cm above ground level, imitating the position of the head of a child sitting on a chair. A video resolution setting of 8 K 3D at 30 fps was used in the recordings. The video material was compiled and edited for the application, resulting in six looping 360-degree videos from different nature sceneries with a minimum loop length of three minutes. Final versions were encoded with H.265 codec, using 7680 × 3840 resolution and a bitrate of approximately 100 Mbps. These videos were used to create the visual aspects of the virtual natural environments. Figure 3 presents the five different virtual natural environments used in the study with the default point of view.

### 3.5. Sound Design of the Virtual Natural Environments

The audio material from the recorded videos was unusable due to distortion caused by the wind and generally low quality. Therefore, a single looping stereo sound file consisting of high-quality and gentle Finnish nature sounds was used for all virtual natural environments. This sound file was originally used and tested in an early prototype of the VirNE application [31]. It is played as a standard stereo file while playing the 360-degree videos, meaning that it does not react to the user’s head movements, unlike the visual environment.

During the development and early testing of this research version of the VirNE application, it was noted that the users did not appear to be bothered by the fact that the nature background sounds were missing the water element visually present in two of these environments, nor did the users seem to be bothered by the fact that the nature sounds were not spatial. This, along with limited resources and the positive results reached in the study with the early prototype [31], encouraged us to use this approach regarding nature sounds in the application, although it is plausible that a high-quality 3D spatial sound design for each environment could improve the immersive qualities of the experience.

### 3.6. Operating the VirNE Application

VirNE application uses Oculus Link for remote control of a Meta Quest 2 HMD. Meta Quest 2 needs a user-defined play area as a safety function, and a room-scale boundary setup was used in this research. After the play area is defined and the Oculus Link connection is established, the application can be started from the computer. In the initial state, the main menu is visible on the computer screen and the HMD shows a still 360-degree picture of clouds and a horizon. In this research, these preparations were conducted by nurses before the arrival of research participants.

**Figure 4:**
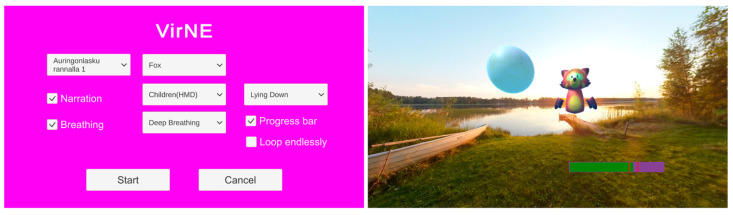
The (**left**) screenshot shows the main menu on the computer screen, which is used to set up the exercise according to the research protocol and patient selections. The (**right**) screenshot presents the computer view during the exercise. The progress bar in the bottom right of the picture is not visible in the HMD view.

During the pre-procedure phase of this study, a research nurse prepared the exercise from the main menu with variables regarding the environment and avatar character the patient selected. When everything was ready, the deep breathing exercise started. Figure 4 presents the computer screen in two different stages of the research.

During the exercise (VR intervention and cannulation phase of the research) the computer screen shows a live feed from the HMD, along with a progress bar indicating the progress of the exercise and a designated timeslot for conducting the medical procedure.

## 4. Materials and Methods of the User Study

A feasibility study was conducted in a local hospital with the VirNE deep breathing exercise with 21 Finnish child patients aged 8–12 years in order to analyze the applicability of the method studied for addressing treatment anxiety in pediatrics. This section presents a short overview of the research protocol, including only information relevant to the user experience viewpoints of the study. The protocol is presented in more detail in the article regarding the effects of the deep breathing exercise [9].

### 4.1. Research Methods

The research protocol of this study was adapted from previous user experience research involving similar research methods and HMD-meditated VR relaxation applications [46]. The protocol is based on evaluating both subjective and objective data regarding the effect of the method studied, both from user experience and medical points of view.

The study participants were child patients due to receive intravenous cannulation as a part of their normal treatment. The intravenous catheter was needed for administering intravenous medication for treatment purposes or for deep anesthesia required for medical imaging. These voluntary participants were chosen based on medical criteria and contacted in person on the day of their scheduled treatment in the hospital, and they used the deep breathing exercise with HMD during the cannulation procedure related to their treatment. However, data related to patient diagnosis is excluded from the scope of this article.

**Figure 5:**
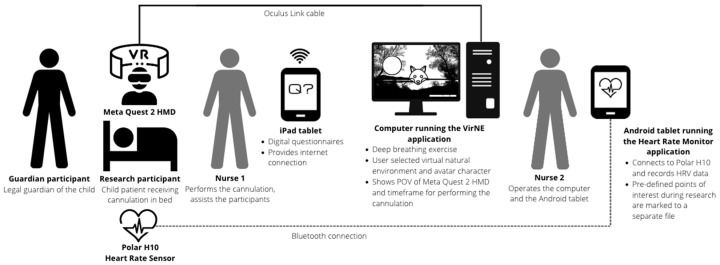
Research scenario. The child patient receiving the treatment is lying on a bed in a half-sitting posture, wearing a Polar H10 chest strap (Polar Electro Oy, Kempele, Finland), and is using a Meta Quest 2 HMD to experience the VR intervention during the cannulation procedure.

The study had a within-subjects design, and it aimed to reduce treatment anxiety during cannulation through the use of the VirNE deep breathing exercise. Guardians of the children participated by providing background information and filling out an anxiety-related questionnaire. Nurses operated the equipment used in the research and performed the cannulation. Figure 5 presents the research scenario.

The study employed a three-phase process. HRV data from participants was collected during all phases but is excluded from the scope of this research article, but the measuring method is presented in the figure for context.

The pre-procedure phase involved clinical measurements, connecting equipment, a structured background interview, and questionnaire responses. Participants also chose the virtual environment and avatar character from physical photos for the VR exercise before wearing the headset.

The VR intervention and cannulation phase included the VirNE deep breathing exercise and the cannulation procedure, which was conducted during a pre-determined timeframe in sync with the deep breathing exercise. After the exercise, the headset was removed and the post-procedure phase began.

The post-procedure phase included a brief unstructured interview, questionnaires including a customized user experience questionnaire, and clinical measurements. After these, the nurses conducting the research concluded the last data collection phase.

### 4.2. The Pediatric Team Used in the Research

The research was conducted by a team of five nurses and a pediatrician. During all research situations, two members of this team were present. It should be noted that medical staff, such as nurses, do not necessarily have a strong technical background. To counter this, the team received hands-on technical training from the authors before and during the initial testing of the research protocol. In addition, the team received a detailed technical operation manual specifically prepared for this research and could contact the authors regarding any possible technical problems during the research.

Additional data for this research article was later acquired through a formal interview of the head of the pediatric team, which was conducted in 2023 after all research participants had participated in the study. This structured interview consisted of 23 questions related to verifying some details regarding the research protocol and observations and information regarding the user experience of the technical equipment used in the research from the viewpoint of pediatric personnel.

### 4.3. Measures

This section of the article provides information about the measures used in this research article. Only data relevant to this article is presented, which excludes most of the medical data gathered during the research. Furthermore, data regarding the effects of the VR intervention on treatment anxiety is excluded but is available in another article by the authors [9]. All questionnaires used in the research were digital Finnish language versions prepared with Microsoft Forms, and research participants entered the data with a tablet during the research situation.

#### 4.3.1. SCARED Questionnaire (Screen for Child Anxiety-Related Emotional Disorders)

The SCARED questionnaire is used to screen symptoms linked to anxiety disorders and phobias [47]. The questionnaire comprises 41 claims concerning unpleasant emotions in various everyday life situations, accompanied by a question of whether the subject relates to the claims constantly, occasionally, or never. These claims can be targeted either directly to the child or to the adult guardian of the child. In this research, both methods were used in the pre-procedure phase. The child participant answered the SCARED for Children questionnaire, while the legal guardian of this child answered the SCARED for Parents questionnaire. The aim was to provide information regarding the possible effect of the evaluated level of anxiety on other data measured in this research.

#### 4.3.2. Customized User Experience Questionnaire

This research used a customized user experience questionnaire in the post-procedure phase to gather data about the user experience of child patients using this stress-reduction method. As the research participants were eight to twelve-year-old children, the questionnaire was intended to be easy to understand and quick to fill in order to keep all participants focused enough to gather reliable research data.

For this reason, a customized four-point user experience questionnaire was prepared, which used a Likert scare (0–6) with 0 labeled as “completely disagree” and 6 as “completely agree”. The questions used in this questionnaire were the following: Q1: “It was easy for me to adjust to being in VR”; Q2: “It was easy for me to focus to the exercise”; Q3: “The application was helpful/useful for me”; and Q4: ”The application was boring for me”.

This questionnaire was, in essence, a highly simplified version of the user experience evaluation methods used in the previous research [46], which used SUXES and UEQ+ questionnaires to gather similar data.

#### 4.3.3. Measures from Interviews

The following measures were derived from the participant interview data, in addition to the more ambiguous information: the level of needle phobia and general user experience. These measures are evaluations from the interview data, conducted by the main authors of this article.

In this study, the level of needle phobia [48] is measured with a Likert scale (0–2). A value of zero equals no needle phobia, whereas a value of one refers to minor signs of needle phobia and a value of two refers to clear signs of needle phobia. This data was obtained from the structured pre-procedure interviews with participants and their legal guardians, who answered a question about experiences regarding needle phobia. This data is used to evaluate the possible effect of needle phobia on the other results.

Furthermore, the general user experience is a measure with three possible values for the experience per participant: positive, neutral, and negative. This data was evaluated from the unstructured post-procedure interviews, and it is used to support the analysis of other variables in the study.

### 4.4. Research Ethics Approval

This study was approved by the Authority Ethics Committee of Pirkanmaa Wellfare Area (R21068L) and the Finnish Medicines Agency (2021/007366).

## 5. Results

A total of 21 child patients and their legal guardians participated in this research. However, data from two participants were excluded from the statistical results after being evaluated to be clear outliers—these two cases include a participant who fainted in the post-procedure phase and had prior experience from fainting during the same medical procedure and a participant who was unable or unwilling to focus on the VR content and constantly tried to look at the real surroundings from the narrow gap between the face and bottom of the HMD. Demographics of the 19 child participants included in the results are presented in Table 2.

The first attempt to cannulate failed with three participants. These participants were asked whether they wanted to continue using the VR intervention or remove the headset before the second attempt, and all three participants wanted to continue with the VR intervention and are included in the results.

### 5.1. VirNE Avatar Character and Virtual Natural Environment Selection

Out of the 19 child participants included in the results, 10 selected the Fox character, while four selected the Bot and five opted to use the application without an avatar character. The mean age (SD) of the participants who selected the Fox character was 9.60 (±1.26), whereas it was 10.25 (±0.96) for the Bot selection, and 10.60 (±1.14) for those who did not want to use an avatar character in the application. Kruskal–Wallis H tests indicated no statistically significant differences between age and avatar selection. While the mean ages suggest a slightly increased preference for no visual avatar character in the exercise among older participants, the number of participants in this study is too low to confirm this.

Regarding virtual natural environment selection, seven participants selected Environment A: *Sunset by a lake shore*, and seven participants chose Environment B: *Ocean shore*, while three selected Environment E: *Coniferous forest*, and one selected Environment C: *Dry coniferous forest*. No participants selected Environment D: *Path in a deciduous forest*. For one participant, data regarding the environment selection was missing.

**Figure 6:**
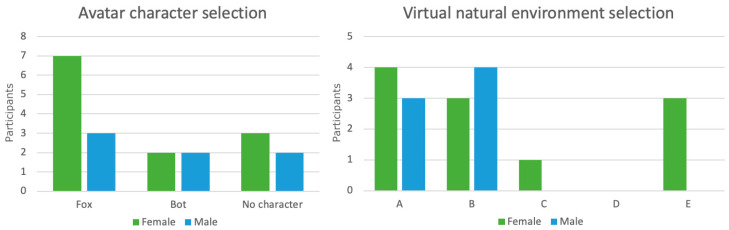
VirNE avatar character and virtual natural environment selections grouped by gender. Environment A is *Sunset by a lake shore*, B is *Ocean shore*, C is *Dry coniferous forest*, D is *Path in a deciduous forest*, and E is *Coniferous forest*.

Figure 6 presents these selections grouped by gender. To summarize, the Fox character was more popular than the other options, and especially favored by female participants, but all options were selected by both genders. Regarding virtual natural environment selection, environments with a water element were popular among the participants, and male participants selected only those two environments.

### 5.2. SCARED Questionnaire (Screen for Child Anxiety-Related Emotional Disorders)

**Figure 7:**
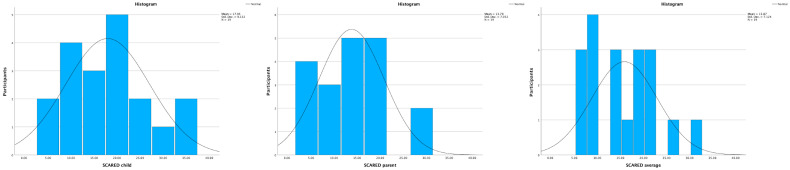
SCARED questionnaire results with histograms and a line presenting normal distribution. The histogram charts represent the sum of participants per total score. All three graphs indicate a non-normal distribution of data.

The SCARED questionnaire results include 19 participants and their legal guardians. The median total scores (IQR) for children and their legal guardians were 18.0 (14.0) and 13.0 (10.0), respectively. To address these differences, a decision was made to use the average values of these two questionnaires when comparing the level of anxiety measured by SCARED questionnaires to the other variables in this study. The median value (IQR) for this SCARED average was 15.0 (12.0). A total score of 25 or more may indicate the presence of an anxiety disorder [47]. As illustrated in Figure 7, the participants of this study can be evaluated to be diverse in terms of levels of anxiety but somewhat skewed towards low anxiety. A more detailed analysis of the SCARED questionnaire results is available in another article by the authors [9].

### 5.3. Customized User Experience Questionnaire

**Figure 8:**
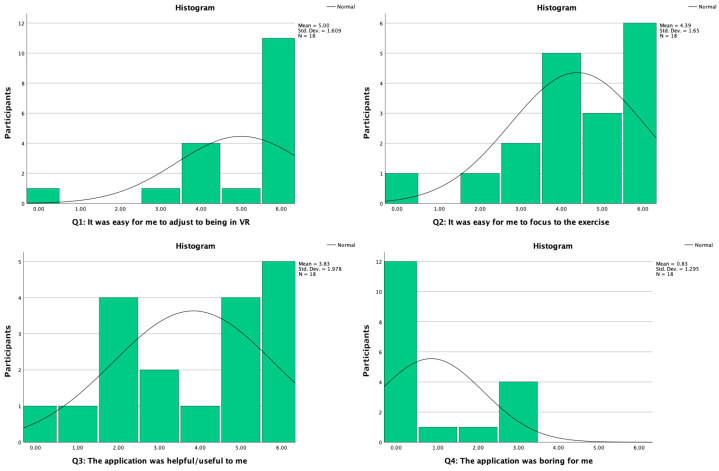
Customized user experience results. The histogram charts represent the sum of participants per each value regarding the question, where a value of six indicates participants “completely agree” with the statement and a value of zero indicates participants “completely disagree”, along with a line presenting normal distribution. All graphs indicate a non-normal distribution of data.

These questionnaire results include only 18 participants, as one participant was excluded due to highly conflicting data in comparison to interview results and user input error was estimated to be very likely. The median values (IQR) for the four questions in this questionnaire were as follows. Q1: *It was easy for me to adjust to being in VR* had a value of 6.00 (2.00), while Q2: *It was easy for me to focus on the exercise* scored 4.50 (2.25). Q3: *The application was helpful/useful to me* had a median value of 4.50 (4.00), and finally Q4: *The application was boring for me* scoring 0.00 (2.25). Figure 8 shows the visual presentation of the results with question-specific histograms.

Q1 and Q4 results strongly suggest that the VR experience was well received, while Q2 results imply that it was relatively easy to focus on the exercise, although it should be noted that about half of the participants seem to have faced at least some problems in focusing. Q3 results are more diverse across the scale, and while being on the positive side on average, six participants rated this question below a value of three, which suggests poor usefulness to one-third of the participants.

### 5.4. Interviews

Unstructured post-procedure interviews of the participants suggest a positive experience for 16 participants, while comments from two participants can be considered as neutral and one participant expressed a negative experience, expressing discomfort from the heart rate sensor and disinterest towards VR technology. Ten participants stated willingness to use the VR method again in the future, while one participant was unsure about it, and one stated being uninterested in any such methods. Eight participants would recommend this method to their friends. Words used to describe the experience include fun (5), helpful (5), good (4), relaxing (2), easy (2), and boring (1).

*“I felt that the exercise helped with the nervousness during the procedure. The exercise was easy to do, and I understood everything.”*

(Participant, 10 years)

*“I liked the character and the scenery, I could do it again.”*

(Participant, 8 years)

Eight participants mentioned liking the virtual natural environment, while one participant expressed a generally positive experience regarding the visuals. Two participants stated liking the avatar character used, while one noted that the mouth of the fox character did not move. Regarding the sound, two participants mentioned liking the sounds in general, while one participant would have preferred a different dialog sound.

*“It was pretty nice. Proper sounds and good landscape. Would recommend to a friend and could use it again too.”*

(Participant, 10 years)

Six participants commented on the effect regarding cannulation. Five of these were positive comments, with four stating they did not notice or feel the needle puncture, one stating the cannulation didn’t bother the participant, and one stating that the VR method did not help with the cannulation although the experience was fun. Finally, no participants reported symptoms of cybersickness [28].

Furthermore, the head of the pediatric team estimated in a structured interview that the effectiveness of the VR intervention was strongly correlated to how well the patient was able to focus on the exercise. Regarding their own user experience, this VR method was estimated to be easy to use, partially due to prior training and written instructions, but the use of the Oculus Link cable was troublesome and was estimated to cause a health security risk both for the patients and the nurses. Furthermore, in normal pediatric treatment, one nurse could plausibly operate the equipment and conduct the procedure—the second nurse was mainly needed due to the HRV measurement.

### 5.5. Data Grouping Analysis

To gain further insight into the data collected, the 19 participants were divided into subgroups according to the following data: prior cannulations, VR experience, breathing exercise experience, level of needle phobia, and gender. These groups were compared regarding data from interviews and customized user experience (UX Q1–4). If a group included a participant who was excluded from the customized user experience results, that participant was excluded from those variables but still counted as a member of the group. However, the interview data is excluded from the statistical analysis and is presented to support the analysis of other variables.

The group division according to prior cannulations is presented in Table 3. Mann–Whitney U tests were conducted to determine whether there were differences between the two groups divided by the amount of prior cannulation experience. The results of these analyses indicated no statistically significant difference regarding the analyzed variables. However, the difference in UX Q3 was on the verge of being statistically significant (z = −2.030, *p* = 0.050), and in UX Q4 it was relatively close to statistical significance (z = 2.172, *p* = 0.077). In other words, the perceived usefulness of the VR intervention was higher among the less experienced patients.

The group division according to previous VR experience is presented in Table 4. Mann–Whitney U tests indicated that variable UX Q4 was lower for those with no prior VR experience (z = −1.017, *p* = 0.034), but no statistically significant difference was found from other variables. In other words, patients with previous VR experience tended to find the VR application less interesting.

The group division according to previous experience with breathing exercises can be seen in Table 5. Mann–Whitney U tests found no statistically significant difference between the groups from any of the analyzed variables. However, it is noteworthy that all participants with prior breathing exercise experience expressed a positive experience in this study, whereas the results were more diverse among the participants without prior experience. In addition, UX questionnaire results seem to be slightly more positive in the group with prior experience. This all indicates a positive effect of previous deep breathing experience on user experience.

The group division according to the estimated level of needle phobia can be found in Table 6. Kruskal–Wallis H tests indicated no statistically significant differences within the analyzed variables. However, the UX results imply a possible positive correlation between the amount of needle phobia and user experience, especially regarding the perceived usefulness of the VR intervention (Q3). Furthermore, it is noteworthy that all participants with clear indications of needle phobia expressed a positive experience.

Finally, when grouped by gender, out of the 12 female participants, 10 expressed a positive experience while two expressed a neutral experience. Out of the seven male participants, six expressed a positive experience and one expressed a negative experience. Mann–Whitney U tests found no statistically significant difference from any of the analyzed variables.

### 5.6. Correlations

The SCARED average data was compared to data from the customized user experience using two-tailed Spearman’s rank–order correlation. A statistically significant moderate positive correlation was found from Q3: *The application was helpful/useful to me*, (*r_s_*(16) *=* 0.519, *p =* 0.027). This means that participants with higher anxiety scores tended to find the VR intervention more helpful than participants with lower anxiety scores.

In addition, the estimated level of needle phobia was compared to data from the customized user experience using two-tailed Spearman’s rank-order correlation. The level of needle phobia had a statistically significant low positive correlation with Q3 (*r_s_*(16) *=* 0.485, *p =* 0.041), and Q2 (*r_s_*(16) *=* 0.448, *p* = 0.062) was relatively close to a statistically significant low positive correlation. This suggests that patients with higher levels of needle phobia found the VR intervention more useful and could also focus on the breathing exercise slightly better.

## 6. Discussion

### 6.1. Main Findings

The data gathered in this research points towards a good user experience among both child patients and pediatricians, with subjective patient evaluations indicating ease in adapting to being in a VR environment and a positive and interesting user experience with no adverse effects. This supports the findings of previous studies regarding the general applicability of HMD-based VR intervention methods in pediatrics for pain and anxiety reduction [2,3,4,5,6]. The safety concerns raised by the nurses conducting the research regarding the use of Oculus Link cable can be solved with wireless remote control methods such as Oculus Air Link [49], although possible negative effects on the technical performance of the VR application need to be considered.

While the article regarding the effect of this method [9] found strong evidence of the stress-reducing effect of the method studied from HRV data across the study population, subjective experiences of the effect were more mixed. According to the user experience data, this is due to both the level of general anxiety and the level of needle phobia positively correlating with how useful the patients evaluated the studied method to be for them. While the mean results for usefulness are positive, they have a large distribution. In other words, patients with an increased need for anxiety reduction found the method most useful.

Furthermore, participants found it relatively easy to focus on the deep breathing exercise, but the data regarding this did vary somewhat between the patients, with results showing subtle signs of improved ability or willingness to focus on the exercise among the patients with higher levels of needle phobia. According to research nurses, focus on the exercise was among the key factors in determining the usefulness of the VR intervention. This indicates a need for interesting and engaging VR content for the child patient, in addition to the therapeutic qualities of the application. In addition, previous experience regarding breathing exercises seemed to positively affect the user experience, suggesting reusability of the method studied.

Interestingly, no indications of cybersickness [28] were found in the study. We estimate that the lack of movement regarding both the patient and the VR intervention contents plays a crucial role here, as cybersickness is related to the subjective feeling of balance and sufficient match between the user movements and the sensory feedback. The combination of using realistic and stable 360-degree videos with no camera movement for a visual VR environment, no user movement within the virtual environment (in addition to the head movements of the user), and a stable half-sitting user posture on a bed seems plausible to support the feeling of physical balance. Furthermore, the relatively short exposure time to VR is likely to minimize the possibility of simulator sickness [50].

### 6.2. Avatar Character and Virtual Natural Environment Selections

Regarding user-selected variables in the application, a clear preference for a water element in a virtual natural environment can be seen from the results across the participants. This supports the findings by Yin et al. [37] regarding the benefits of cultural context in virtual natural environments, as children in Finland are often very familiar with water elements. Finland has many inland lakes and a relatively long ocean shoreline, and a day at a beach is a popular pastime among Finnish families during summer months and is thus easily associated with positive experiences.

However, some participants did prefer forest environments, which are also common in Finland. Even so, no participants selected the environment D: *Path in a deciduous forest*. Some of the possible affective qualities regarding this environment include that it can be estimated to be the least common environment in Finland from the available selections and that it includes a clear human element in the form of a path. Furthermore, it can also be evaluated to be relatively dark in brightness in comparison to other available virtual natural environments, which is a factor that has been suggested to affect the stress reduction potential of virtual natural environments [51].

In addition, some gender-related differences could be found. The selections of female participants were more diverse, and male participants seemed to strongly prefer a water element. However, the number of participants is too low for definite conclusions. In general, the findings of this study support the idea of using 360-degree videos of local natural environments with no camera movement for virtual natural environments, and the use of several different options to select from can be recommended for adjusting to individual preferences.

Similarly, all three avatar character options were selected by the participants, with about a quarter of participants opting to use no avatar character in the application. While selections were diverse among both genders, the Fox character seems to be more favored by female participants. Regarding age, older patients selected the no avatar character option more often than the patients from the younger side of the spectrum. However, as with virtual natural environments, no definite conclusions should be drawn due to the relatively small sample size and diversity of possible affective factors. Even so, the use of several different options with a proper focus on the target user group seems to be a plausible design for a good user experience.

### 6.3. Previous Studies

We were unable to find previous studies regarding the pediatric use of HMDs for deep breathing exercises in virtual natural environments. However, Ferraz-Torres et al. [4] recently studied a similar scenario, where 124 pediatric patients with a mean age of 8.4 years (±4.1 SD) used an HMD to experience interactive or passive VR content while undergoing venipuncture for blood extraction or vascular cannulation. This study used vector graphics-based virtual natural environments for both forms of VR content, which can be less realistic and thus potentially less immersive than 360-degree videos of natural locations.

Their findings suggest that HMD-based VR methods are beneficial in pediatric care as a distraction method, but interestingly also claim that experiencing a passive VR environment is inferior to interactive VR experience in anxiety reduction in invasive pediatrics, with results suggesting that 88.9% of the patients using passive VR experienced anxiety during the procedure. However, the study groups in the study were uneven regarding the age difference and number of participants, with the passive VR group (*n* = 36) having a very low mean age of 3.8 years (±1.6 SD); therefore, participant age may have affected the results concerning the benefits of the VR intervention the participants used.

Even so, their results are in partial contradiction with our findings, as the method we studied found positive results with relatively passive VR content in terms of direct user interaction, although the breathing balloon animation can be considered as an interactive element on top of the more passive elements in the VirNE application used in our study.

Xiang et al. [3] also compared passive and interactive VR contents in pediatrics in their study regarding the use of smartphone-based VR with patients suffering from burn pain, with the results suggesting the inferiority of passive VR content in pediatric anxiety and pain management. However, their study used a VR game for both active and passive VR content, with the difference being the ability to interact with the game—in other words, the passive VR content was content designed for interaction but without the possibility of interaction.

Another study with a similar approach and results was conducted by Piskorz et al. [52], who also used a game for active VR content, and the main difference to passive VR content was the ability to interact. We estimate this kind of approach to be likely to favor active VR content.

Encouraged by our findings, we argue that a more accurate approach to evaluate the potential of passive VR content in pediatrics would include content that is designed for passive use. Such was the case in a study by Gerçeker et al. [6], which compared two different VR contents to a control group during blood drawing. Both VR contents they used can be evaluated to be passive in terms of interaction, as one group experienced a rollercoaster ride in VR with the primary aim of distracting the patient, and the other group used more relaxing content, namely, an underwater tour with marine animals and soft music in VR. Both approaches resulted in reduced pain and anxiety in comparison to the control group. Interestingly, there was no significant difference in the effect size regarding pain, but the relaxing VR content produced a larger decrease in anxiety.

While direct interaction with the VR application can increase user engagement in VR with positive results, especially regarding patient distraction from the medical procedure, we suggest that pediatric VR content can also have a more passive approach, such as one using relaxing scenery and guided relaxation exercises, which can also distract, although in a more subtle manner. However, rather than focusing on the amount of interaction, we suggest an alternative and plausibly more meaningful way to approach the design of VR content for pediatric use, which is a design process based on applying methods from existing knowledge regarding the desired effect, the target user group, and the use context.

In our case, such digital conversions of traditional affective methods included the positive mental health effects of being in natural surroundings [8,30] and the stress-reducing effect of conscious deep breathing [7,38]. In addition, the audiovisual design of the VR application was targeted at children to improve focus and engagement, and the functionality was designed for pediatric use, including support for remote control, a design for a half-sitting user posture, and a deep breathing exercise design which included a pre-determined point for the invasive medical procedure. This all resulted in an application that is relatively passive but does include some interactive elements.

Prabhu et al. [53] conducted a recent study on a very similar VR method involving 30 older adults to address surgical anxiety and pain management in both preoperative and postoperative settings. In their research, they incorporated a deep breathing exercise in a virtual natural environment and measured HRV data. However, their study went a step further by employing a more advanced VR application design that integrated biofeedback and gamification elements for guidance and motivation. This randomized control trial employed a between-subjects design, assigning participants to control, 2D video (laptop computer screen), or VR (HMD) groups. Both the 2D video and VR groups exhibited reduced pain and anxiety coupled with increased parasympathetic activity compared to the control group. Notably, the VR group demonstrated the most significant decrease in pain. This reinforces the use of deep breathing exercises in virtual natural environments in pediatrics as well. In addition, it highlights the potential benefits of incorporating biofeedback and gamification to enhance the design of such VR relaxation applications. Furthermore, while their findings indicate the superiority of VR methods to 2D screen-based implementations, the possibility of using the latter option could be beneficial in some use cases.

Finally, as Simonetti et al. [5] recommended in their recent systematic review and meta-analysis of the field, and based on our experiences and outcomes of this research, we also recommend VR product development for pediatrics to be performed in true multidisciplinary collaboration between pediatricians and professional VR developers.

### 6.4. Implications and Recommendations

The use of guided deep breathing exercises in virtual natural environments is an effective and pleasant non-pharmaceutical method to address treatment anxiety in pediatrics. Therefore, this method shows strong potential to improve patient satisfaction in invasive procedures such as cannulation while also supporting the consistency of the treatment. Further research with larger study samples and child patients suffering from treatment anxiety is highly recommended.

The positive results of this study offer a proof-of-concept regarding the multidisciplinary design approach when developing VR applications for pediatrics, with the designed application being not only effective but also providing a good user experience among both the patients and pediatric personnel operating the application. Furthermore, as the patient’s focus on the VR content is essential, the pediatric VR content needs to be not only theoretically effective and designed for pediatric use but also to provide an interesting and engaging experience for the child patient. Good user experience design and a multidisciplinary design team can help achieve these results.

Biofeedback and gamification elements are recognized as potential directions for further development of this VR relaxation method. These elements could plausibly further improve patient engagement with the VR application.

In addition, the results reached with this design approach challenge the idea of evaluating the usefulness of a VR method in pediatrics based on the amount of patient interaction with the application. We recommend further research regarding VR methods that address treatment anxiety in pediatrics to be conducted with VR applications specifically designed for stress reduction and targeted for children, instead of choosing VR methods based on the amount of interaction.

The use of 360-degree videos of local nature environments recorded with a still camera stand proved to be a suitable and cost-effective method for creating stress-reducing virtual natural environments. We recommend using at least 8 K resolution 360-degree videos of sunny natural environments with local cultural context and minimal visible or audible human influence. It is also noteworthy that forest environments are not the only option for good results, and that the soundtrack of the video can and should be replaced with a more suitable one if needed.

### 6.5. Limitations

The study population was diverse but relatively small, and it had relatively low anxiety levels in comparison to the general population. As the method studied aims to reduce anxiety levels, more research with a larger study population would be needed regarding the applicability of this method in pediatrics, especially regarding high anxiety levels.

Information regarding the patient diagnosis and the treatment path related to intravenous cannulation is excluded from the scope of this article. It needs to be noted that some medical conditions can negatively affect the usefulness or user experience of this method.

No control or comparison groups were used in this study, which limits the applicability of these results. Further research with a comparison group is needed before the user experience and perceived usefulness of this VR relaxation method can be reliably compared to other VR methods in pediatrics. Possible excitement from experiencing novel VR technology cannot be separated from the other factors affecting the user experience in this study.

In addition, these results provide no information beyond speculation on how repeated use of the method would affect the user experience.

## 7. Conclusions

The results of this study support the idea of using VR with HMDs in pediatric care, and more precisely deep breathing exercises in virtual natural environments, with the technology being well-received among both the child patients and pediatricians involved in this study. Over three-quarters of the participants reported a positive experience, with fun and helpful being the two most common words used to describe the experience.

The perceived usefulness of this method correlates with the levels of general anxiety and needle phobia of the patient. This, along with the stress-reducing effect of the method, demonstrates high usefulness for addressing treatment anxiety. More research with child patients suffering from treatment anxiety is therefore highly recommended.

A multidisciplinary design approach based on existing scientific knowledge regarding the desired effect is likely to be superior to more abstract design approaches that focus on the level of interaction in the pediatric use of VR.

Finally, the use of stationary 360-degree videos from peaceful natural locations with local cultural context and minimal visible or audible human influence is an effective approach when designing virtual natural environments for stress reduction.

## Figures and Tables

**Table 1:** VirNE deep breathing exercise structure.

Timeframe	Exercise Phase Description	Dialog	Breathing Animation	Marked for Procedure
0:00–0:30	The selected virtual natural environment starts to play, and the selected avatar character is present	**-**	**-**	**-**
0:30–1:00	The exercise starts. Introduction to the exercise and setting the body posture	**X**	**-**	**-**
1:00–2:00	Teaching conscious slow breathing. Breathing animation appears	**X**	**X**	**-**
2:00–	Guided slow deep breathing for 30 breathing cycles of six seconds starts. Short encouraging dialog cues are played periodically, once per 30 s	**X**	**X**	**-**
3:30–4:00	The period marked for conducting the cannulation, visible only to the nurse controlling the application with the PC	**-**	**X**	**X**
–5:00	Guided slow deep breathing continues until 5:00	**X**	**X**	**-**
5:00–5:30	Returning to normal breathing and ending the exercise. The breathing animation disappears	**X**	**-**	**-**
5:30–6:00	The virtual environment continues to play for 30 more seconds. This period aims for a smooth ending of the experience, and the removal of the head-mounted display is conducted during it	**-**	**-**	**-**

**Table 2:** Demographics of the child patients included in the statistical results.

Data Group	Study Sample
*n*	19 participants
Age (mean (SD))	10.1 (±1.29) years
Gender division	12 females, 7 males
Height (mean (SD))/weight (mean (SD))	144.0 (±11.8) cm/38.2 (±10.5) kg
Prior intravenous cannulations *0–2 cannulations* *3+ cannulations*	*10 participants* *9 participants*
Level of needle phobia *0—no needle phobia* *1—minor needle phobia* *2—clear needle phobia*	*7 participants* *6 participants* *6 participants*
Prior virtual reality experience	11 had experience, 8 did not
Prior deep breathing experience	11 had experience, 8 did not
Sensitivity to motion sickness *low/medium/high*	*9/6/4 (participants)*

**Table 3:** Group division according to prior cannulations.

Data Group	0–2 Prior Cannulations	3+ Prior Cannulations
*n*	10	9
Interview (UX): pos./neut./neg.	9/0/1	7/2/0
UX Q1 mean (SD)	4.67 (±2.00)	5.33 (±1.12)
UX Q2 mean (SD)	4.56 (±1.13)	4.22 (±2.11)
UX Q3 mean (SD)	4.78 (±1.48)	2.89 (±2.02)
UX Q4 mean (SD)	0.11 (±0.34)	1.56 (±1.51)

**Table 4:** Group division according to prior VR experience.

Data Group	VR Experience: No	VR Experience
*n*	8	11
Interview (UX): pos./neut./neg.	7/0/1	9/2/0
UX Q1 mean (SD)	5.13 (±2.10)	4.90 (±1.20)
UX Q2 mean (SD)	4.63 (±1.30)	4.20 (±1.93)
UX Q3 mean (SD)	4.00 (±2.00)	3.70 (±2.06)
UX Q4 mean (SD)	0.00 (±0.00)	1.50 (±1.43)

**Table 5:** Group division according to prior deep breathing experience.

Data Group	Deep Breathing Experience: No	Deep Breathing Experience: Yes
*n*	8	11
Interview (UX): pos./neut./neg.	5/2/1	11/0/0
UX Q1 mean (SD)	4.71 (±2.21)	5.18 (±1.17)
UX Q2 mean (SD)	3.86 (±2.04)	4.73 (±1.35)
UX Q3 mean (SD)	3.00 (±1.83)	4.36 (±1.96)
UX Q4 mean (SD)	0.86 (±1.46)	0.82 (±1.25)

**Table 6:** Group division according to the estimated level of needle phobia.

Data Group	0 = No Needle Phobia	1 = Minor Needle Phobia	2 = Clear Needle Phobia
*n*	7	6	6
Interview (UX): pos./neut./neg.	6/1/0	4/1/1	6/0/0
UX Q1 mean (SD)	5.14 (±1.21)	4.40 (±2.61)	5.33 (±1.03)
UX Q2 mean (SD)	3.57 (±1.99)	4.40 (±1.52)	5.33 (±0.82)
UX Q3 mean (SD)	2.86 (±2.34)	3.40 (±1.52)	5.33 (±0.82)
UX Q4 mean (SD)	1.29 (±1.60)	1.00 (±1.41)	0.17 (±0.41)

## Data Availability

Summary of the data presented in this study is contained within the article. Full dataset not available due to ethical and privacy issues.
